# Comparative analysis of adaptive immunity to SARS-CoV-2 in infected children and adults

**DOI:** 10.1038/s41390-025-04256-x

**Published:** 2025-08-20

**Authors:** Sabryna Nantel, Corey Arnold, Maala Bhatt, Yannick Galipeau, Benoîte Bourdin, Jennifer Bowes, Roger L. Zemek, Marc-André Langlois, Caroline Quach, Hélène Decaluwe, Anne Pham-Huy

**Affiliations:** 1 Sainte-Justine University Hospital and Research Center, Montreal, QC Canada; 2 Department of Microbiology, Infectious Diseases and Immunology, Faculty of Medicine, University of Montreal, Montreal, QC Canada; 3 Department of Biochemistry, Microbiology and Immunology, Faculty of Medicine, University of Ottawa, Ottawa, ON Canada; 4 Children’s Hospital of Eastern Ontario Research Institute, University of Ottawa, Ottawa, ON Canada; 5 Division of Emergency Medicine, Department of Pediatrics, University of Ottawa, Ottawa, ON Canada; 6 Pediatric Immunology and Rheumatology Division, Department of Pediatrics, University of Montréal, Montréal, QC Canada; 7 Division of Infectious Diseases, Immunology and Allergy, Department of Pediatrics, Children’s Hospital of Eastern Ontario, University of Ottawa, Ottawa, ON Canada

## Abstract

**Background:**

SARS-CoV-2 infection in children is most often mild and resembles that of seasonal coronaviruses. Profiling the adaptive immune response following infection may help to inform on the protective mechanisms mediating immunity in children and adults.

**Methods:**

Humoral and cell-mediated immune responses from unvaccinated pediatric and adult participants were analyzed following non-Omicron SARS-CoV-2 infection. Specific T cell memory responses were investigated by quantifying interferon-gamma (IFN-γ) secreting cells after stimulation with ancestral and variant strains of SARS-CoV-2 and seasonal human β-coronaviruses (HCoV)-OC43 and -HKU1.

**Results:**

Twenty-eight children (3–17 [median = 10] years) and 28 adults (19–62 [median = 42] years) were sampled at a mean time of 7 months (±2.8 months) after SARS-CoV-2 infection. Antibody levels against spike (S) and the receptor-binding domain (RBD), as well as neutralization capacity, were equivalent in adults and children. However, children displayed a lower number of IFN-γ secreting T cells in response to SARS-CoV-2 compared to adults, with a median of 88 [28–184] spot-forming units (SFU) per million of cells in children compared to 208 [141–340] in adults (*P* < 0.001). In children, the IFN-γ^+^ responses to SARS-CoV-2 were of similar magnitude as the responses to seasonal β-coronaviruses (*P* > 0.05). In contrast, adults exhibited heightened T cell responses to SARS-CoV-2 than they did to HCoV-OC43 (median of 80 [45–135] SFU/10^6^ cells, *P* < 0.0001) and HCoV-HKU1 (median of 98 [59–151] SFU/10^6^ cells, *P* < 0.01).

**Conclusions:**

In children, the functional T cell memory responses to SARS-CoV-2, assessed through IFN-γ secretion in response to peptide stimulation, are comparable to those of HCoVs and lower compared to adults.

**Impact:**

- While binding and neutralizing antibody levels to SARS-CoV-2 are largely comparable in children and adults, the strength of memory T cell responses induced by infection is reduced in children compared to adults.
- Adults present heightened cellular memory responses to SARS-CoV-2, but not to seasonal β-coronaviruses HCoV-OC43 and HCoV-HKU1.
- In contrast, children’s T cell memory responses to SARS-CoV-2 closely mirror their response to common seasonal β-coronaviruses.

## Introduction

Since the beginning of the coronavirus disease 2019 (COVID-19) pandemic, there have been age-related differences in clinical presentations and outcomes of severe acute respiratory syndrome coronavirus 2 (SARS-CoV-2) infection. Severe disease disproportionately impacts older adults, in contrast to children, who generally have mild or asymptomatic disease.^1–3^ Several factors could account for these differences, such as fewer co-morbidities in children, some degree of immunity conferred by prior exposure to seasonal coronaviruses, or reduced expression of angiotensin-converting enzyme 2 (ACE2) in children, leading to reduced viral replication.^4–8^ In addition, there are increasing reports of differences noted in the host response to SARS-CoV-2 in children compared to adults.^9,10^ Studies describe children’s host response to SARS-CoV-2 to be characterized by a more robust, albeit transient, innate immune response.^11^ Previous studies have already demonstrated the production of neutralizing antibodies (nAb) following SARS-CoV-2 infection in children, even in mild or asymptomatic infection.^12,13^ Studies focusing on antibody responses to the spike and its receptor-binding domain (RBD) showed increased binding and nAb levels in children compared to adults, especially in young children under 5 years old.^14,15^ As cell-mediated immunity is more complex to study, less is known about the characteristics of T cells that develop in children. Knowledge gaps remain, especially regarding the persistence of T cell immunity more than 6 months after infection and its contribution to immune memory and risk of reinfection in children.^16–19^ As the pandemic ended, there was a decrease in the perceived need to vaccinate children. COVID-19 vaccination rates in those aged from 6 months to 4 years remain very low, therefore highlighting the need to better understand the adaptive immunity developed following infection in children.^20,21^ The goal of this study was to describe and compare antibody and T cell responses following SARS-CoV-2 infection in children and adults and to contrast them with the response elicited by seasonal coronaviruses.

## Methods

### Study design and data collection

This was a multicenter, longitudinal study, combining two existing prospective cohorts. Pediatric participants were recruited from the *Persistence of Antibody Titers to COVID-19 in Households (PATCH Study)*, a case-ascertained antibody-surveillance initiative based in Ottawa, Ontario.^22^ Children or adolescents with confirmed SARS-CoV-2 infection defined by either positive SARS-CoV-2 polymerase chain reaction (PCR) testing or positive anti-nucleocapsid (anti-N) SARS-CoV-2 immunoglobulin G (IgG) from the PATCH cohort were eligible to participate. Twenty-eight participants had samples that were suitable for T cell analysis. Adult participants were selected from the *RE-Infection in COVID-19 Estimation of Risk Cohort (RECOVER Study)*, which consisted of health care workers who were recruited following PCR-confirmed SARS-CoV-2 infection (*N* = 569).^23,24^ Adult samples were matched to the pediatric samples based on sex, time since infection, and ethnicity when the information was disclosed by the participants. All participants were unvaccinated. Our study included one time point per participant, which was around 7 months post-infection. Questionnaires documented demographics, medical history, COVID-19 symptoms, and potential recent or recurrent SARS-CoV-2 infection.^22–24^

### Sample collection and processing

Blood samples from the pediatric cohort were collected at the Children’s Hospital of Eastern Ontario (CHEO) and cryopreserved at the Coronavirus Variants Rapid Response Network Biobank within 4 h (and up to 24 h) of procurement, while those from the adult cohort were processed and cryopreserved at the Mother Child Biobank at the Sainte-Justine University Hospital and Research Center (CR-CHUSJ). Serum and peripheral blood mononuclear cells (PBMCs) were isolated for the assessment of antibody levels and T cell studies, respectively. This was done according to standard operating procedures (SOPs) using SepMate^TM^ tubes (Stemcell Technologies, Canada). PBMCs were resuspended in complete Roswell Park Memorial Institute (RPMI) media (Gibco) with 10% dimethyl sulfoxide (DMSO) and cryopreserved in liquid nitrogen, while serum aliquots were cryopreserved at −80 °C. Frozen samples from both cohorts were sent and analyzed using the same ELISpot assay (Dr. Decaluwe’s laboratory), binding antibody and neutralization assays (University of Ottawa’s Serology and Diagnostics High-Throughput Facility, Dr. Langlois’ laboratory). Both pediatric and adult samples were processed at the same time using the same assays. Each sample was tested for quality and viability before proceeding with T cell assays. Of the 38 pediatric samples collected, 28 had a sufficient number of cells with adequate viability to proceed with the ELISpot assay. Subsequently, each of the pediatric samples was matched to one adult control from the RECOVER cohort.

### Date of infection

The date of infection was determined by the earliest of either (1) the onset of symptoms or (2) the confirmed testing date. For asymptomatic pediatric individuals who were never tested but had confirmed infection through the presence of anti-N IgG, the infection date was estimated based on their infected household member (confirmed by PCR).

### Study period and SARS-CoV-2 variant circulation

Blood samples were collected from May to December 2021. The initial SARS-CoV-2 infections occurred between December 2020 and April 2021, before the emergence of the Omicron (BA.1) variant. No participants reported symptoms suggestive of a new infection at the time of blood collection. Specific testing to identify SARS-CoV-2 variants was not conducted. Instead, the likely strain of SARS-CoV-2 was determined based on prevalent local epidemiology. Infections from March 2020 to March 2021 were most likely caused by the ancestral (Wuhan-like) strain. In contrast, infections from March 1, 2021 to April 2021 were attributed to the Alpha (B.1.1.7) variant, as this variant was predominant in Ontario between March 1 and July 31, 2021.^25^

### Humoral immunity

#### Chemiluminescent direct enzyme-linked immunosorbent assay (ELISA) for antibody detection

Serum samples were analyzed using automated chemiluminescent ELISAs for SARS-CoV-2-specific IgA and IgG targeting the whole spike (S) protein, the receptor binding domain of S (RBD), and the nucleocapsid (N) protein. Binding antibodies were all assessed for the ancestral (Wuhan-like) strain. This analysis was conducted with the Hamilton MicroLab STAR robotic liquid handlers at the University of Ottawa’s Serology and Diagnostics High-Throughput Facility (Faculty of Medicine), as previously described.^26^ Briefly, diluted serum samples were incubated in 384-well assay plates (Thermo Fisher Scientific, Waltham, MA) coated with 50 ng/well of S, RBD, or N (S and N from NRC Metrology Division, RBD from the laboratory of Dr. Yves Durocher). Secondary horseradish peroxidase (HRP)-conjugated antibodies against IgG and IgA were added to the wells and detected following incubation using SuperSignal Pico chemiluminescent substrate (Thermo Fisher Scientific, Waltham, MA). Plate reader raw luminescence values were measured, blank-subtracted, and scaled to an on-plate standard curve. Scaled values were then converted to international binding antibody units per mL (BAU/mL) based on the World Health Organization (WHO) international standard (NIBSC code 20/136) and to lab-specific units (µg/mL) using established four-parameter logarithmic conversion models.^22^ For each antigen (S, RBD, and N), a cut-off was established based on false discovery rates in a pre-pandemic cohort (3%, 2%, and 5%, respectively).^26,27^ Seropositive status at analysis was defined as IgG binding to the whole spike over the cut-off based on false discovery rates in the pre-pandemic cohort, which was 15.53216 BAU/mL.

#### Chemiluminescent surrogate neutralization ELISA (snELISA) for neutralizing antibody detection

snELISA assesses the capacity of serum samples to inhibit the interaction between the S protein of SARS-CoV-2 and the human ACE2 cell receptor.^22^ In all, 384-well assay plates were coated with 100 ng/well of trimeric S protein for ancestral SARS-CoV-2 (Wuhan, NRC Metrology Division) or the Omicron variant strains BA.1 and BA.4/5 (from the laboratory of Dr. Yves Durocher). Samples were serially diluted to generate five data points and incubated on the assay plates. Following incubation, 6.5 ng of biotinylated human ACE2 (NRC Metrology Division, Nova Scotia, Canada) was incubated in each well and subsequently detected using a streptavidin-peroxidase polymer (Thermo Fisher Scientific, Waltham, MA) and SuperSignal Pico chemiluminescent substrate (Thermo Fisher Scientific, Waltham, MA). Luminescence readings were background-subtracted, and each dilution point was expressed as a percentage of the average signal from wells with uninhibited antigen–ACE2 interaction (maximum signal). Three-parameter logarithmic regression was applied to model the % of inhibition as a function of the dilution factor, with fixed upper and lower points at 100% and 0%, respectively. The inhibitory dilution at 50% inhibition (ID50) for each sample was determined from its regression and presented as the reciprocal dilution factor (an ID50 of 100 indicates a sample diluted 1:100 has the capacity to inhibit 50% of ACE2–antigen binding).

### Cell-mediated immunity

#### Enzyme-linked immunospot (ELISpot) for the quantification of functional cellular immunity

Interferon-gamma (IFN-γ)-secreting cells were detected through the ELISpot assay. After rapid thawing and overnight resting, PBMCs were stimulated with 1 µg/mL of S, N, or membrane (VME1) mega pools of SARS-CoV-2 peptides from the ancestral strain, as well as the spike from variants B.1.1.7 (Alpha), B.1.351 (Beta), P.1 (Gamma), B.1.617.2 (Delta), and B.1.1.529 (Omicron) and from two other β-coronaviruses (HCoV-OC43 and HCoV-HKU1) (JPT Peptide Technologies, JPT, Berlin, Germany) as described elsewhere.^28^ Culture media without peptide (AIM-V® Medium (1×), Thermo Fisher Scientific, Waltham, MA) and CytoStim^TM^ (Miltenyi Biotec, MA) were used as negative and positive controls, respectively. Spots were quantified using CTL ImmunoSpot® SS UV Analyzer (Cellular Technology Ltd., OH). The positive threshold response was defined as 25 spot-forming units (SFU) per million of PBMCs, as previously published by our team.^23,29^

### Statistics

Mann–Whitney or Kruskal–Wallis unpaired nonparametric tests were performed using Prism 9, version 9.2.0 (2021 GraphPad Software, LLC) to assess statistical significance. Significance was set as *P < 0.05, **P < 0.01, ***P < 0.001 and ****P < 0.0001. Time since infection is presented as mean ± SD, while experimental data are displayed as median [interquartile range, 25th–75th percentile].

## Results

### Participant characteristics

There were 28 children (median age = 10 years, [Min–Max], [3–17], 50% female) and 28 adults (42 years [19–62], 50% female) included in this study for which samples were suitable for analysis (Table 1). One time point was analyzed per study participant, with a mean time since infection of 7.4 ± 2.8 months for children and 7.0 ± 2.5 months for adults. Most children reported mild COVID-19 symptoms, categorized as WHO scale 2–3, similar to the adult group, with the exception of four children who were asymptomatic and identified through household exposure history (Table 1). Due to the timing of the studies and delayed recruitment of infected children compared to adults, 57% (*N* = 16) of children were presumed to have been infected while the B.1.1.7 Alpha variant was predominant in Ontario (Table 1). In contrast, all adults were presumably infected by the ancestral Wuhan-like strain.

**Table 1:** Characteristics of participants included in the analysis.

Characteristics	Number (%) of children (*n* = 28)	Number (%) of adults (*n* = 28)
*Age (years)*		
Median [Min, Max]	10 [3, 17]	42 [19, 62]
*Sex*		
Male	14 (50)	14 (50)
Female	14 (50)	14 (50)
*Race or ethnicity*		
White	15 (54)	17 (61)
Middle Eastern	3 (11)	3 (11)
Asian	8 (29)	8 (29)
Undisclosed	2 (7)	0 (0)
*Primary SARS-CoV-2 infection*		
Asymptomatic—WHO Score 1	4 (14)	0 (0)
Symptomatic (mild)—WHO Score 2–3	24 (86)	28 (100)
Severe/hospitalized—WHO Score 4–5	0 (0)	0 (0)
*Presumed strain based on infection period*		
Ancestral (Wuhan-1 like)	12 (43)	28 (100)
Alpha variant	16 (57)	0 (0)
*Time since infection at analysis (months)*		
Mean (SD)	7.4 (2.8)	7.0 (2.5)
Median [Min, Max]	7.3 [2.7, 15.4]	7.2 [2.7, 15.4]
*Serology status at analysis*^a^		
Seronegative	2 (7)	1 (4)
Asymptomatic	1 (4)	0 (0)
Symptomatic	1 (4)	1 (4)
Seropositive	26 (93)	27 (96)
Asymptomatic	3 (11)	0 (0)
Symptomatic	23 (82)	26 (93)

^a^Serology status was defined as IgG binding to the whole spike over the cut-off value.

### The strain of infection minimally influenced the level of adaptive immunity in children

We first ascertained the potential differences induced by the divergent infection strains in the pediatric participants, to control for the influence of the strain of infection when later comparing the pediatric to the adult cohort, infected by the Wuhan-like ancestral virus. Serum IgG and IgA antibody levels specific to the SARS-CoV-2 ancestral (Wuhan-like) S protein, RBD, and N were quantified. When comparing humoral responses in children presumably infected by the ancestral (Wuhan-like) strain or B.1.1.7 Alpha variant, both groups showed comparable IgG and IgA responses across all three antigens, with no statistically significant difference (Fig. S1A, B). However, nAb against the ancestral strain were slightly increased in children infected with the B.1.1.7 Alpha variant compared to those infected with the ancestral strain, while they were comparable when neutralizing Omicron BA.1 or BA.4/0.5 variants (Fig. S1C). When assessing the cellular immune response to the spike, membrane, or nucleocapsid peptides, or to multiple SARS-CoV-2 spike variants, no significant differences were observed in the number of IFN-γ-secreting T cells whether children were presumably infected by the ancestral or Alpha variant (Fig. S1D, E).

### Children showed comparable levels of anti-spike antibodies but reduced anti-nucleocapsid antibodies when compared to adults

**Fig. 1:**
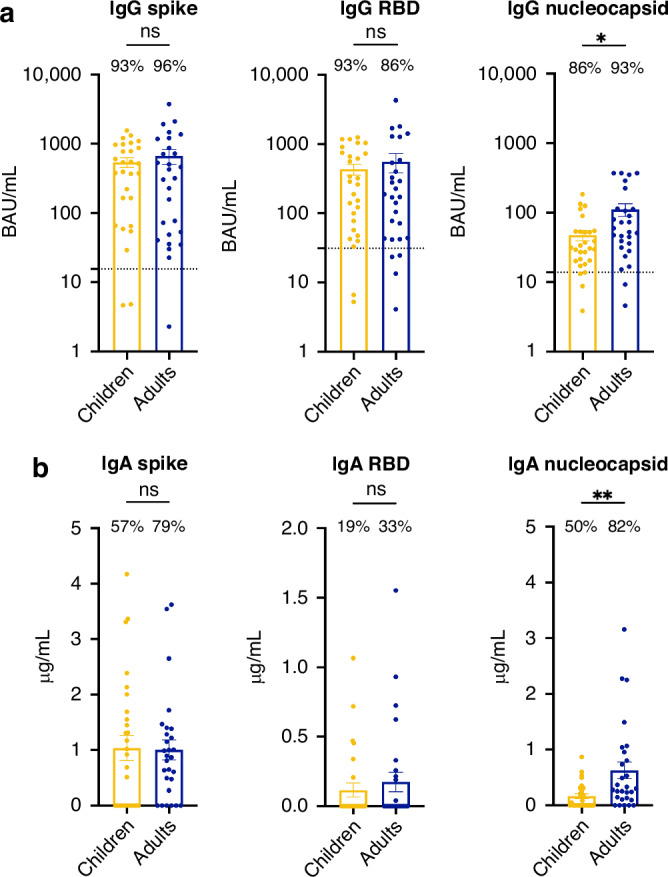
Children exhibited lower antibody levels against non-spike components of SARS-CoV-2 compared to adults. ELISA was conducted to measure the anti-spike (left panels), anti-RBD (middle panels) and anti-nucleocapsid (right panels) IgG (**a**) and IgA (**b**) levels in the serum of children (*N* = 28, yellow) and adults (*N* = 28, blue) infected with SARS-CoV-2. **a** The dotted lines indicate the positive cut-off value of 15.53 for the spike, 31.35 for the RBD and 13.84 for the nucleocapsid. The percentage of participants with IgG responses above these cut-offs is shown for each group. **b** The percentage of participants with detectable IgA is indicated for each group. Error bars indicate mean ± SEM. Statistical significance was established as not significant (ns) *P* > 0.05, *P < 0.05, **P < 0.01.

As the strain of infection marginally influenced the adaptive immune response in children, we then interrogated and compared the memory immune response induced by SARS-CoV-2 infection between the pediatric and adult cohorts. SARS-CoV-2 seropositivity was defined using SARS-CoV-2 spike-specific IgG ELISA in the serum (Table 1). Most subjects were seropositive (93% of children and 96% of adults) at the time of sampling. Children and adults had comparable levels of anti-S and anti-RBD antibodies, but children had lower antibody levels against the nucleocapsid protein, which is one of the non-spike components of SARS-CoV-2 (Fig. 1). Notably, both IgG (Fig. 1a) and IgA (Fig. 1b) binding to the nucleocapsid protein were significantly lower in children with a median of 31.0 [20.2–53.8] BAU/mL in children compared to 56.1 [32.6–113.8] BAU/mL in adults (*P < 0.05) for IgG and 0.02 [0.00–0.31] µg/mL in children compared to 0.29 [0.11–0.92] µg/mL in adults (**P < 0.01) for IgA. Because we previously reported that symptomatology and serology status affected the strength of the immune response in infected adults, we questioned whether it may influence results in the pediatric population as well.^23^ When asymptomatic (*N* = 4 children) and seronegative (*N* = 1 child and 1 adult) participants were excluded from the analysis, the difference in IgA levels against the nucleocapsid remained, while it was less evident for anti-nucleocapsid IgG levels (Fig. S2).

### Children and adults exhibited comparable neutralizing capacity

**Fig. 2:**
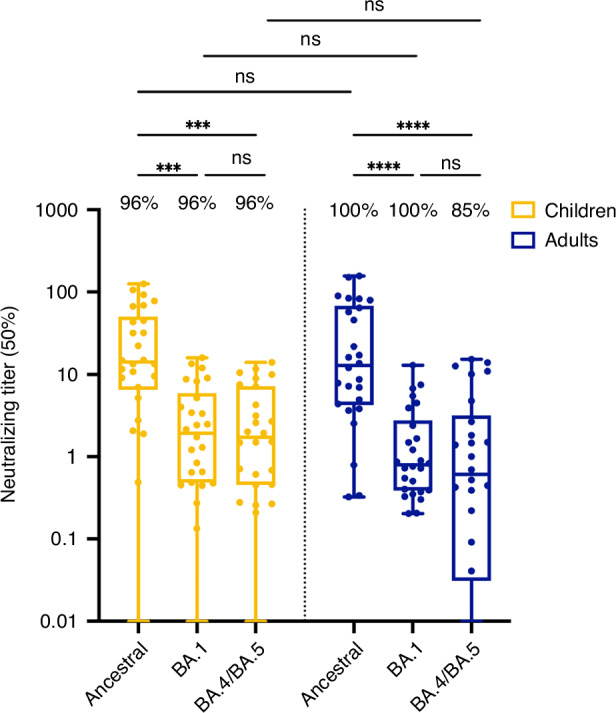
Children and adults developed similar antibody neutralizing capacity to ancestral SARS-CoV-2 and Omicron variants. Surrogate neutralization ELISA was conducted to establish the serum dilution level (ID50) required to inhibit 50% of the binding between the trimeric spike protein and the ACE2 receptor, thus neutralizing the virus attachment capability to cells. Neutralizing antibody titers were measured for the ancestral SARS-CoV-2 spike, as well as Omicron BA.1 and BA.4/BA.5 variants. Serums were analyzed after SARS-CoV-2 infection in children (*N* = 26, yellow) and adults (*N* = 26, blue). The percentage of participants with detectable neutralizing antibodies against specific variants is indicated for each group. Statistical significance was established as not significant (ns) *P* > 0.05, ***P < 0.001, ****P < 0.0001.

Blockade of SARS-CoV-2 viral entry through the ACE2 receptor is largely mediated by Igs targeting the spike protein and its RBD component.^30–32^ Neutralizing capacity was assessed through surrogate neutralization ELISA against the ancestral SARS-CoV-2 as well as Omicron variants BA.1 and BA.4/BA.5. Children and adults had similar neutralizing capacity against all three strains assessed. As demonstrated in other studies, neutralizing capacity decreased against newer variants (Omicron BA.1 and BA.4/BA.5) compared to the ancestral strain, a finding similar in both populations (Fig. 2).^33^ We then excluded children and adults who either had an asymptomatic infection or were seronegative at inclusion. We noted comparable nAb levels for the ancestral strain, but slightly increased nAb titers against Omicron BA.1 and Omicron BA.4/BA.5 in children, compared to adults (*P < 0.05; Fig. S3).

### The strength of T cell response to SARS-CoV-2 was reduced in children compared to adults

**Fig. 3:**
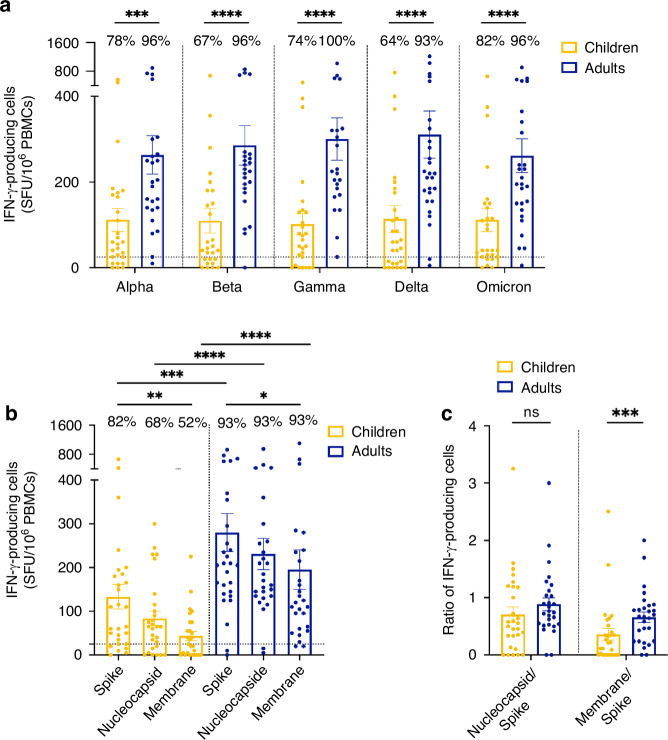
Cellular immune responses to SARS-CoV-2 were enhanced in adults compared to children. T cell responses were assessed by ELISpot assay after peptide stimulation. Samples were collected from SARS-CoV-2-infected children (*N* = 28, yellow) and adults (*N* = 28, blue). **a** PBMCs were stimulated with SARS-CoV-2 spike peptide from five different variant strains (Alpha, Beta, Gamma, Delta, Omicron BA.1). **b** PBMCs were stimulated with peptide pools from the ancestral SARS-CoV-2 spike, nucleocapsid and membrane protein. **c** Ratio of IFN-γ-producing cells per million PBMCs in response to the nucleocapsid and the membrane protein relative to the response to the spike. Results are expressed in the number of IFN-γ-producing cells per million PBMCs. The dotted lines indicate the positive threshold value of 25 IFN-γ-secreting cells. The percentage of participants with responses above this threshold is indicated for each group and condition. Error bars indicate mean ± SEM. Statistical significance was established as not significant (ns), not shown on graphs; *P* > 0.05, *P < 0.05, **P < 0.01, ***P < 0.001, ****P < 0.0001.

Adaptive cellular immunity against SARS-CoV-2 is characterized by IFN-γ production from T cells, which serves as an important indicator of their antiviral activity.^17,34^ To assess the functional capacity of memory T cells after SARS-CoV-2 infection, ELISpot assays were performed to quantify IFN-γ-secreting cells following stimulation with various SARS-CoV-2 peptide pools. For all SARS-CoV-2 strains tested, children displayed less IFN-γ-secreting cells compared to adults (Fig. 3a). The number of antigen-responding T cells per million PBMCs was twofold lower in children compared to adults, with a median of 88 [28–184] SFU per million of PBMCs in children compared to 208 [141–340] in adults (***P < 0.001) for the ancestral Wuhan-like spike (Fig. 3a). Similar results were also observed for the Alpha, Beta, Gamma, Delta, and Omicron BA.1 strains. Children showed significantly lower IFN-γ responses to all three viral components of the Wuhan-like strain (the spike (***P < 0.001), the nucleocapsid (****P < 0.0001), and the membrane protein (****P < 0.0001)) when compared to adults (Fig. 3b). The reduced magnitude of the T cell response in children was observed even when individuals who had an asymptomatic infection or were seronegative at analysis were excluded (Fig. S4). We further assessed within-group immunodominance by calculating the ratio of IFN-γ-secreting cells in response to non-spike versus spike components of the ancestral virus for each participant. Children and adults showed comparable nucleocapsid to spike ratios with mean ratios of 0.70 and 0.89, respectively, while children showed significantly reduced membrane to spike ratios compared to adults, with mean ratios of 0.36 and 0.66 (***P < 0.001; Fig. 3c).

### The enhanced T cell response in adults is specific to SARS-CoV-2

**Fig. 4:**
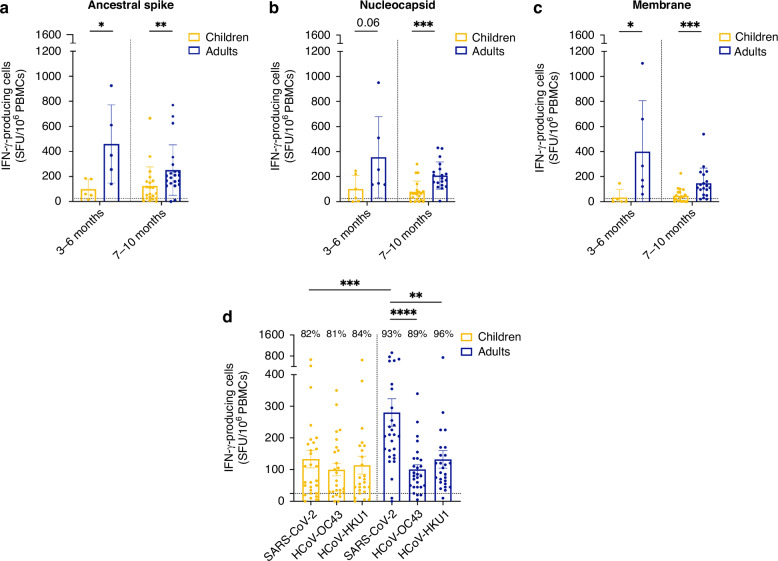
The enhanced T cell response in adults is observed early after infection and is specific to SARS-CoV-2. T cell responses were assessed by ELISpot assay after peptide stimulation. Samples were collected from SARS-CoV-2-infected children (yellow) and adults (blue) at 3–6 months post-infection (*N* = 5) or 7–10 months post-infection (*N* = 21). PBMCs were stimulated with peptide pools from the ancestral SARS-CoV-2 spike (**a**), its nucleocapsid (**b**), and its membrane protein (**c**). **d** PBMCs were stimulated with the spike peptides from SARS-CoV-2 and common cold β-coronaviruses HCoV-OC43 and HCoV-HKU1, respectively. Results are expressed in the number of IFN-γ-producing cells per million PBMCs. The dotted lines indicate the positive threshold value of 25 IFN-γ-secreting cells. **d** The percentage of participants with responses above this threshold is indicated for each group and condition. Error bars indicate mean ± SEM. Statistical significance was established as not significant (ns), not shown on graphs; *P* > 0.05, *P < 0.05, **P < 0.01, ***P < 0.001, ****P < 0.0001.

To better understand the differences observed between children and adults in terms of their T cell immune response to SARS-CoV-2 peptides, we first interrogated the impact of time on the T cell IFN-γ response. We divided our cohorts based on the time since infection at blood sampling and compared the number of IFN-γ-secreting cells at early (3–6 months) and late (7–10 months) time points after infection in both cohorts, excluding the two samples drawn more than 1 year after the initial infection (Fig. 4). We showed that the reduced strength of the T cell responses observed in children was already noticeable in the early phase of the memory response between 3 and 6 months with a median of 60 [15–185] SFU/10^6^ PBMCs in children compared to 370 [140–925] SFU/10^6^ PBMCs in adults for the ancestral spike. The T cell response remained stable at later time points (7–10 months) in children with a median of 65 [0–665] SFU/10^6^ PBMCs, but it reduced to 205 [0–770] SFU/10^6^ PBMCs in adults. While contraction of the T cell response was more pronounced in adults, children exhibited a stable number of IFN-γ-secreting T cells throughout the observation period (Fig. S5). We then questioned if the differences observed between children and adults were specific to SARS-CoV-2, or if children also displayed reduced T cell responses to seasonal β-coronaviruses when compared to adults. We thus assessed and compared the T cell responses to HCoV-OC43 and HCoV-HKU1, in both pediatric and adult cohorts. Unexpectedly, children displayed responses to SARS-CoV-2 of similar strength as they did to HCoV-OC43 and HCoV-HKU1 (median of 88 [28–184] for SARS-CoV-2 compared to 58 [29–163] and 60 [33–168] SFU per million PBMCs for HCoV-OC43 and HCoV-HKU1, respectively, *P* > 0.05). In contrast, adults exhibited robust IFN-γ secretion to SARS-CoV-2 (median of 208 [141–340]), but significantly lower responses to common β-coronaviruses (median of 80 [45–135], ****P < 0.0001 for HCoV-OC43 compared to SARS-CoV-2 and 98 [59–151], **P < 0.01 for HCoV-HKU1 compared to SARS-CoV-2) (Fig. 4d). Importantly, both children and adults T cells responded to seasonal coronaviruses with the same strength (*P* > 0.05, not shown on graphs). Therefore, the increase in IFN-γ-secreting cells observed in adults was specific to SARS-CoV-2. These results suggest that children exhibit T cell immune responses of similar intensity as adults to seasonal coronaviruses, but do not present the same exacerbation of the T cell response to SARS-CoV-2 as adults do.

For all three assays used to evaluate the memory humoral and cellular responses to SARS-CoV-2 infection, no sex-based difference was observed (Fig. S6). Only one graph is shown for each assay, but comparison was done for all conditions without any sex-based difference detected through statistical analyses (Table S1).

## Discussion

This study aimed to contribute to a better understanding of the differences in adaptive immune responses following SARS-CoV-2 infection in children compared to adults. Specifically, the goal of this study was to bridge the knowledge gap regarding T cell immunity in children. Using a cohort of 28 pediatric and 28 adults matched for symptom severity, time since infection at blood draw, sex, and ethnicity, we described the humoral and cellular immune responses following SARS-CoV-2 infection and identified clear distinctions between the two groups. In the convalescence phase of SARS-CoV-2 infection, children and adults exhibited comparable antibody responses, as assessed by their levels of serum Igs and antibody neutralizing capacity. In contrast, adults demonstrated enhanced memory T cell responses compared to children, at both early (3–6 months) and late (7–10 months) time points after infection. Despite lower numbers of SARS-CoV-2-specific T cells identified in children, their functional memory response was comparable to that of other seasonal β-coronaviruses, in striking contrast with the adult cohort. Further, in children, we observed a preferential reduction in the response to the membrane component of the virus.

Increasing number of reports have observed an enhanced T cell responses to SARS-CoV-2 in adults rather than children.^35–38^ Children were also shown to mount an early and effective response to non-spike components of SARS-CoV-2 such as the nucleocapsid that was shown to wane and decline more rapidly than in adults.^9,36,39,40^ T cells have an important role in limiting disease severity, leading to recovery but also to immune memory and protection against reinfection by future variants.^41^ Rapid IFN-γ secretion is associated with efficient and protective anti-viral immunity, which demonstrates the relevance of assessing cell-mediated memory immune responses through this functional IFN-γ secretion assay.^5,6^ Indeed, the capacity of T cells to promptly secrete IFN-γ in response to specific SARS-CoV-2 stimulation is associated with mild disease and accelerated viral clearance.^42^ Reports have shown that the severity of initial SARS-CoV-2 infection impacts the amplitude of the T cell response, with stronger but often impaired and pathogenic responses in individuals with severe disease.^16,17,23^ Our study shows that mild disease led to higher T cell responses to SARS-CoV-2 in adults compared to children. This is consistent with the study by Khoo et al., who described multimodal immune responses, including cellular responses, in a cohort of five children and seven adults in the acute and convalescent phases of infection.^38^ Their results showed that adults generated stronger T cell immune memory responses and that children showed less activation of the T cell compartment.^38^ Our data also concurs with Cohen et al., who also showed that CD4^+^ memory T cell responses to structural SARS-CoV-2 proteins increased with age.^35^ This phenomenon was suggested to be correlated to prior exposure to β-coronaviruses, with adults having a higher lifetime exposure to HCoVs compared to young children.^35,43^ In adults, the repeated exposures to HCoVs likely generate a larger pool of memory T cells that can recognize conserved epitopes between HCoVs and SARS-CoV-2. Stimulating with SARS-CoV-2 peptides may therefore lead to a more robust T cell response in adults, as reflected by higher IFN-γ production. On the other hand, although children may have encountered a HCoV more recently than adults, their pre-existing pool of memory T cells is smaller, as both seasonal coronaviruses and SARS-CoV-2 stimulate mainly naïve T cells.^44^

Our data contrasts with those of Dowell et al., who reported that children mount more robust adaptive humoral and cell-mediated immune responses than adults.^40^ Of note, their pediatric participants were younger than our cohort of mostly school-aged children, which may influence the strength of the adaptive immune response.^45^ Further, recent exposure to β-coronaviruses and elevated levels of HCoV cross-reactivity observed in their cohort could contribute to the differences observed between our studies. It underscores the importance of time since the latest coronavirus infection and the age of the subject when evaluating the magnitude of the adaptive immune response to SARS-CoV-2.

As mentioned above, memory T cells generated following HCoV infections are able to cross-react with SARS-CoV-2 antigens, as demonstrated using pre-pandemic samples, in both adults and children.^43,46,47^ This cross-reactivity is established very early in life.^43^ However, despite numerous HCoV exposures, Humbert et al. showed that the capacity of T cells to cross-react declines with age, as does the capacity of CD4^+^ T cells to respond to HCoVs.^43,47^ In line with this, we reported that the increased level of T cell memory observed in adults is unique to SARS-CoV-2 and is not seen when evaluating responses to seasonal HCoVs, as demonstrated by their lower level of response to HCoV-HKU1 and HCoV-OC43. In our study, children exhibited cellular responses to SARS-CoV-2 comparable to those elicited in response to seasonal β-coronaviruses. This is interesting as the clinical picture of SARS-CoV-2 for the majority of children is also very similar to common cold HCoVs. It may suggest that SARS-CoV-2 leads to inherently distinct immune responses in adults and children, with SARS-CoV-2 being recognized as would other seasonal coronaviruses in children.

The differences between adults and children in the strength of the T cell responses to SARS-CoV-2 could be explained by a rapid and extremely efficient innate response in children. Indeed, it is thought that children have pre-activated and robust innate immune responses that are very effective at controlling and clearing SARS-CoV-2 infection.^11,19^ The study by Vono et al. demonstrated that children mount a more robust and effective innate immune response to SARS-CoV-2 compared to adults.^11^ This response was characterized by higher initial levels of CD14^+^CD16^+^ monocytes, greater upregulation of natural killer cell activity, and increased numbers of plasmacytoid and myeloid dendritic cells during the first 5 days of infection. These findings suggest that children exhibit a more efficient early immune response. In contrast, adults experienced a more prolonged inflammatory response, which may contribute to increased disease severity and immunopathology. Additionally, nasopharyngeal sampling showed that healthy children have in their upper airways immune cells in an IFN-activated state even in the absence of infection, while immune cells were rarely detected in the nasal mucosa of healthy uninfected adults.^19^ Considering the demonstrated susceptibility of SARS-CoV-2 to IFNs, this enhanced viral sensing capacity of airway epithelial cells could limit viral dissemination.^48^ This may greatly impact the adaptive T cell response, as early viral clearance by the innate immune response would restrict antigen availability and the amplitude of long-lived T cell responses.^19,49^ Further, an accelerated innate immune response may lead to a more coordinated and balanced adaptive immune response in children, which could contribute to their milder symptoms and reduced inflammatory complications.

Although there was general homogeneity in the clinical presentation of the cases in both cohorts, there were limitations to our study, mostly related to sampling. The sample size was relatively small, and we were only able to analyze one time point. The time from infection to blood sampling, while matched between the two cohorts, was variable between study participants, ranging from 3 to 15 months post-infection (median of 7 months). The timing of blood sampling was also delayed in the pediatric cohort, which led to half of that cohort being presumably infected with the B.1.1.7 Alpha variant. Although distinct SARS-CoV-2 strains may influence the strength and breadth of the adaptive immune response, the close relationship between the Wuhan-like and the Alpha variant did not significantly influence our results, as demonstrated in this study. Other limitations included viral characterization, which was not included in the study but was inferred from careful epidemiological data. Blood sampling was also limited due to pediatric volume restrictions. Consequently, due to the limited cell numbers, peptide or protein-specific mapping was not possible, nor was the determination of the specific T cell subsets responding to the virus. Although quantification of the functional cell-mediated immune response provides a reductive appreciation of T cell immunity to SARS-CoV-2, it allowed us to investigate responses to multiple variants and distinct components of the virus.

The main strength of our study was the direct comparison of both humoral and cell-mediated immunity following mild SARS-CoV-2 infection in adults and children, while also interrogating responses to seasonal coronaviruses. We showed that T cell responses to SARS-CoV-2 are strikingly elevated in adults compared to responses to HCoV-OC43 and HCoV-HKU1, an analysis that was not performed in previous studies. Further, we discovered that T cell responses to SARS-CoV-2 are comparable to those of other β-coronaviruses in children, which may highlight distinct dynamics of the immune response to respiratory viruses in young children compared to adults. Collectively, our results suggest that in the convalescent phase, T cell responses to SARS-CoV-2 are attenuated in children compared to adults and are similar to those of other common seasonal coronaviruses. Despite studying the host response in the pre-Omicron era and in unvaccinated individuals, this study remains relevant in contributing to a better understanding of pediatric host response dynamics to SARS-CoV-2 and seasonal coronaviruses. Studying the cell-mediated immune responses in children to respiratory viral infections remains important to understand protective mechanisms of prolonged immune protection and risk of reinfection in children.

## Conclusion

Previous studies suggested that robust innate immune responses help to clear SARS-CoV-2 infection rapidly in children, similar to other seasonal coronaviruses. This highly effective clearance of viral antigens may lead to less T cell activation, therefore inducing a distinct T cell differentiation program and dampened IFN-γ^+^ T cell responses in children compared to adults. Further longitudinal studies are needed to establish the diversity and duration of protection mediated by T cells in children and whether vaccination is useful in broadening and increasing the durability of protection against SARS-CoV-2 infection and severe disease in children. For now, our study demonstrated that the pediatric host immune response to SARS-CoV-2 is similar to that of other seasonal coronaviruses.

## Supplementary information

Supplementary Material

## Data Availability

The datasets generated during the current study are available from the corresponding author on request.
