# Supplementary material for: Comparative analysis of adaptive immunity to SARS-CoV-2 in infected children and adults
Source: Pediatr Res. 2025 Aug 20;99(3):1104–12. doi: 10.1038/s41390-025-04256-x (PMC13021522; doi:10.1038/s41390-025-04256-x)
Supplement: Supplementary file 1 — Supplementary Material [file 41390_2025_4256_MOESM1_ESM.pdf]

Supplementary Material for :

Comparative Analysis of Adaptive Immunity to SARS-CoV-2 in Infected Children and Adults

Running Title : : Immunity to SARS-CoV-2 – Just a "common cold" for children

Authors : Sabryna Nantel,[1,2] Corey Arnold,[3] Maala Bhatt,[4,5] Yannick Galipeau,[3] Benoîte Bourdin,[1] Jennifer Bowes,[4] Roger L. Zemek,[4,5] Marc-André Langlois,[3] Caroline Quach,[1,2] Hélène Decaluwe [1,2,6,#,*] & Anne Pham-Huy [4,7,#,*]

Affiliations :

[1] Sainte-Justine University Hospital and Research Center, Montréal, Québec, Canada.

[2] Department of Microbiology, Infectious Diseases and Immunology, Faculty of Medicine, University of Montréal, Montréal, Québec, Canada.

[3] Department of Biochemistry, Microbiology and Immunology, Faculty of Medicine, University of Ottawa, Ottawa, Ontario, Canada.

[4] Children's Hospital of Eastern Ontario Research Institute, University of Ottawa, Ottawa, Ontario, Canada.

[5] Division of Emergency Medicine, Department of Pediatrics, University of Ottawa, Ottawa, Ontario, Canada.

[6] Pediatric Immunology and Rheumatology Division, Department of Pediatrics, University of Montréal, Montréal, Québec, Canada.

[7] Division of Infectious Diseases, Immunology and Allergy, Department of Pediatrics, Children's Hospital of Eastern Ontario, University of Ottawa, Ottawa, Ontario, Canada.

# Shared senior authorships

CORRESPONDING AUTHORS (*)

Dr. Hélène Decaluwe

CHU Sainte-Justine Research Center

3175, Chemin de la Côte-Sainte-Catherine, Montréal, QC, Canada (H3T 1C5)

Dr. Anne Pham-Huy

Children's Hospital of Eastern Ontario

401, Smyth Road, Ottawa, ON, Canada (K1H 8L1)

aphamhuy@cheo.on.ca

SUPPLEMENTAL FIGURES AND LEGENDS

Figure S1. Infections presumably caused by the B.1.1.7 Alpha variant and the ancestral Wuhan-like strain induced comparable adaptive immune responses in children.

Figure S2. The IgA response against the nucleocapsid remained lower in children when removing subjects who were asymptomatic or seronegative.

Figure S3. Children presented slightly increased neutralizing antibody titers to Omicron variants compared to adults after excluding asymptomatic and seronegative individuals.

Figure S4. Reduced cellular immune responses to SARS-CoV-2 in children compared to adults remained after excluding asymptomatic and seronegative indivuals.

Figure S5. Cellular immunity to SARS-CoV-2 was maintained in children but contracted in adults.

Figure S6. Adaptive immune responses to SARS-CoV-2 were comparable in males and females.

SUPPLEMENTAL TABLE

Table S1. Statistical analyses for sex-based comparisons of SARS-CoV-2-specific adaptive immune responses.

Figure S1. Infections presumably caused by the B.1.1.7 Alpha variant and the ancestral Wuhan-like strain induced comparable adaptive immune responses in children

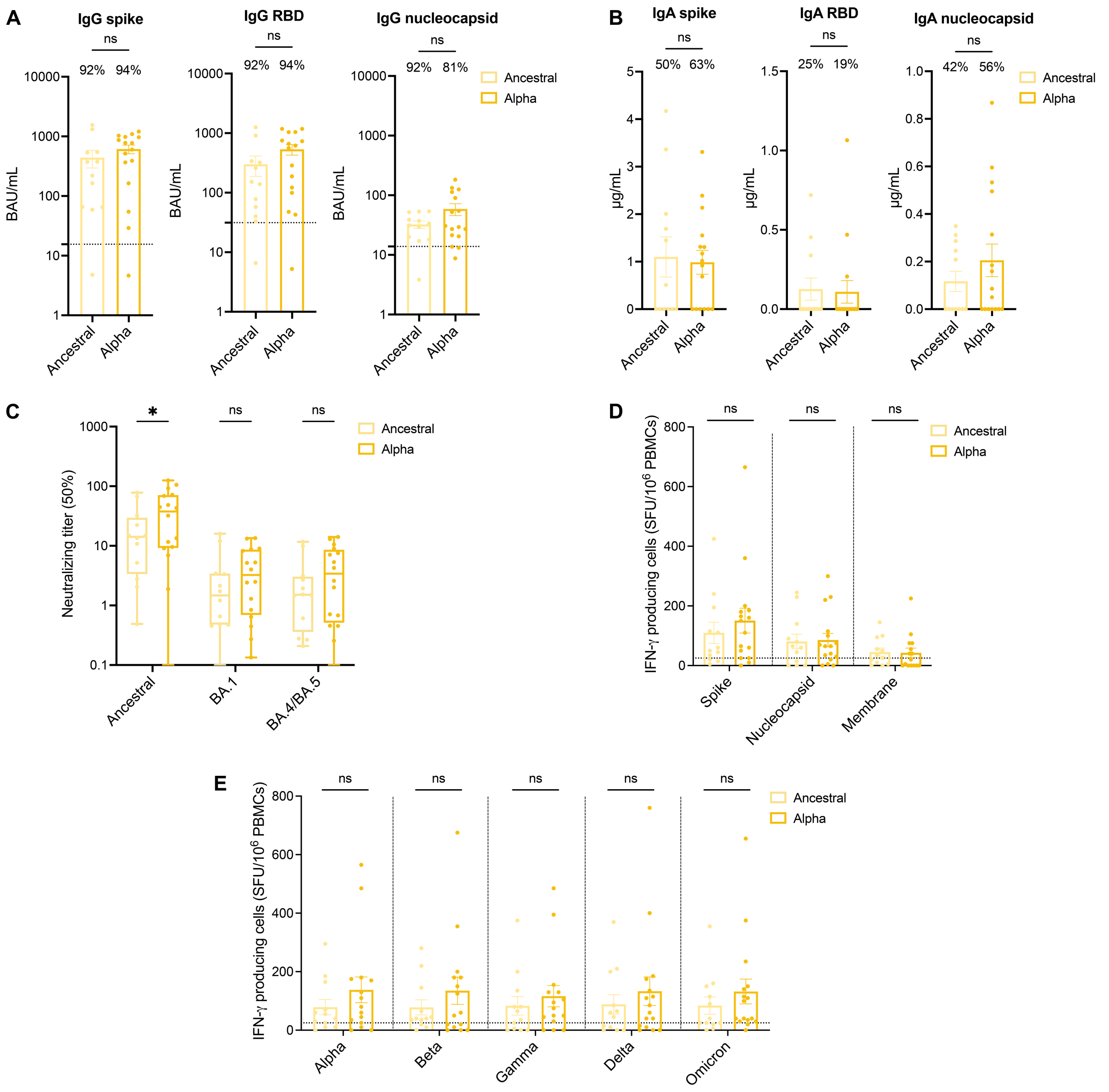

Samples were collected from children presumably infected with the ancestral strain of SARS-CoV-2 (N = 12, light yellow) and children presumably infected with the B.1.1.7 Alpha variant of SARS-CoV-2 (N = 16, dark yellow). (**A-B**) ELISA were conducted to measure the anti-spike **(left panels)**, anti-RBD **(middle panels)** and anti-nucleocapsid **(right panels)** IgG **(A)** and IgA **(B)**

levels in the serum. **(A)** The dotted lines indicate the positive threshold values of 15.53 for the spike, 31.35 for the RBD, and 13.84 for the nucleocapsid. The percentage of participants with IgG responses above these cut-offs are shown for each group. **(B)** The percentage of participants with detectable IgA are indicated for each group. **(C)** Surrogate neutralization ELISA were conducted to establish the serum dilution level (ID50) required to inhibit 50% of the binding between the trimeric spike protein and the ACE-2 receptor, thus neutralizing the virus's attachment capability to cells. Neutralizing antibody titers were measured for the ancestral SARS-CoV-2 spike, as well as Omicron BA.1 and BA.4/BA.5 variants. (**D-E**) T-cell responses were assessed by ELISpot assay after peptide stimulation. **(D)** PBMCs were stimulated with peptide pools from the ancestral SARS-CoV-2 spike, nucleocapsid and membrane protein. **(E)** PBMCs were stimulated with SARS-CoV-2 spike peptide from five different variant strains (Alpha, Beta, Gamma, Delta, Omicron BA.1). Results are expressed in number of IFN-γ producing cells per million PBMCs. The dotted lines indicate the positive threshold value of 25 IFN-γ secreting cells. Error bars indicate mean ± SEM. Statistical significance was established as not significant (ns) $P > .05$, $^*P < .05$.

Figure S2. The IgA response against the nucleocapsid remained lower in children when removing subjects who were asymptomatic or seronegative

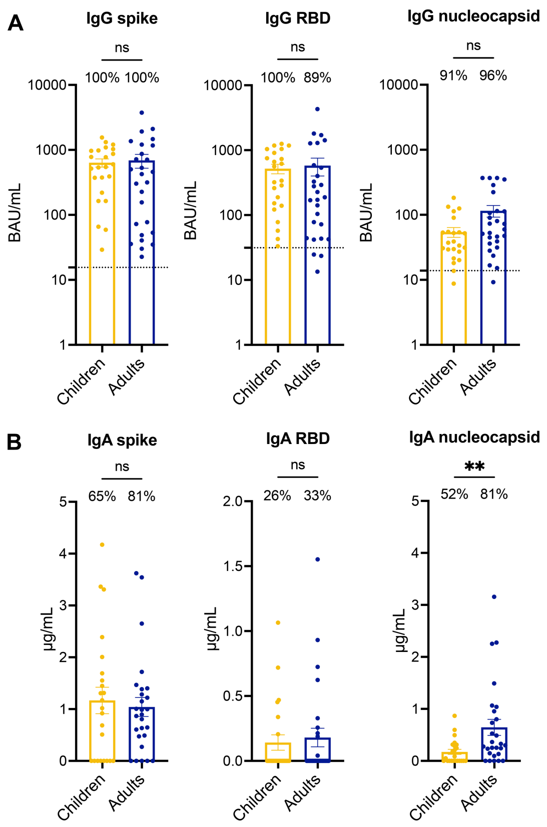

ELISA were conducted to measure the anti-spike **(left panels)**, anti-RBD **(middle panels)** and anti-nucleocapsid **(right panels)** IgG **(A)** and IgA **(B)** levels in the serum of symptomatic and seropositive children (N = 23, yellow) and adults (N = 27, blue) infected with SARS-CoV-2. **(A)** The dotted lines indicate the positive threshold values of 15.53 for the spike, 31.35 for the RBD, and 13.84 for the nucleocapsid. The percentage of participants with IgG responses above these cut-offs are shown for each group. **(B)** The percentage of participants with detectable IgA are indicated for each group. Error bars indicate mean ± SEM. Statistical significance was established as not significant (ns) P >.05, *P <.05.

Figure S3. Children presented slightly increased neutralizing antibody titers to Omicron variants compared to adults after excluding asymptomatic and seronegative individuals

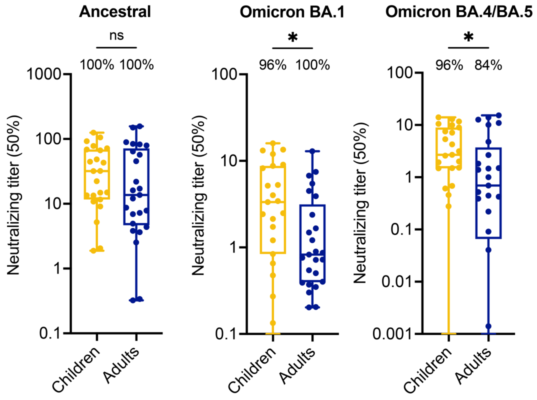

Surrogate neutralization ELISA were conducted to establish the serum dilution level (ID50) required to inhibit 50% of the binding between the trimeric spike protein and the ACE-2 receptor, thus neutralizing the virus's attachment capability to cells. Neutralizing antibody titers were measured for the ancestral SARS-CoV-2 spike, as well as Omicron BA.1 and BA.4/BA.5 variants. Serums were analyzed after SARS-CoV-2 infection in symptomatic and seropositive children (N = 23, yellow) and adults (N = 25, blue). The percentage of participants with detectable neutralizing antibodies against specific variants are indicated for each group. Error bars indicate mean ± SEM. Statistical significance was established as not significant (ns) P >.05, *P <.05.

Figure S4. Reduced cellular immune responses to SARS-CoV-2 in children compared to adults remained after excluding asymptomatic and seronegative indivuals

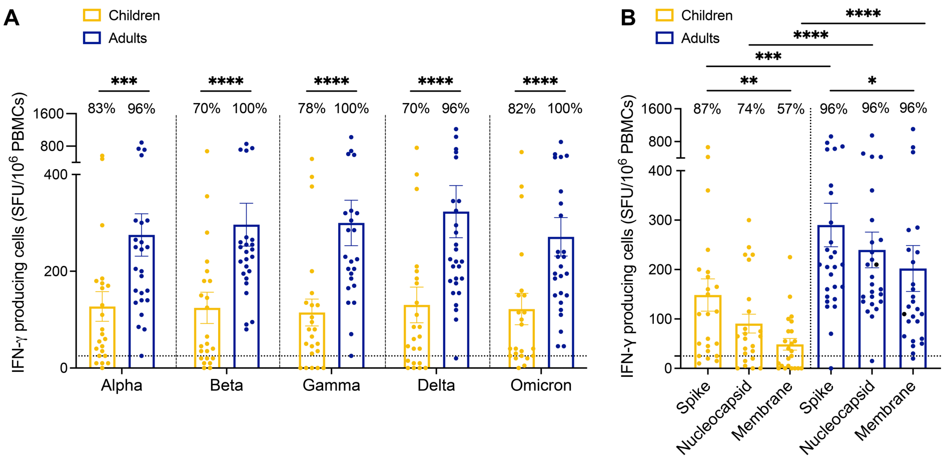

T-cell responses were assessed by ELISpot assay after peptide stimulation. Samples were collected from SARS-CoV-2 infected children who were symptomatic at the time of infection and seropositive at sampling (N = 23, yellow) and adults (N = 27, blue). **(A)** PBMCs were stimulated with SARS-CoV-2 spike peptide from five different variant strains (Alpha, Beta, Gamma, Delta, Omicron BA.1). **(B)** PBMCs were stimulated with peptide pools from the ancestral SARS-CoV-2 spike, nucleocapsid and membrane protein. The dotted line indicate the positive threshold value of 25 IFN-γ secreting cells. The percentage of participants with values above this threshold are shown for each group and condition. Error bars indicate mean ± SEM. Statistical significance was established as not significant (ns), not shown on graphs P >.05, *P <.05, **P <.01, ***P<.001, ****P<.0001.

Figure S5. Cellular immunity to SARS-CoV-2 was maintained in children but contracted in adults

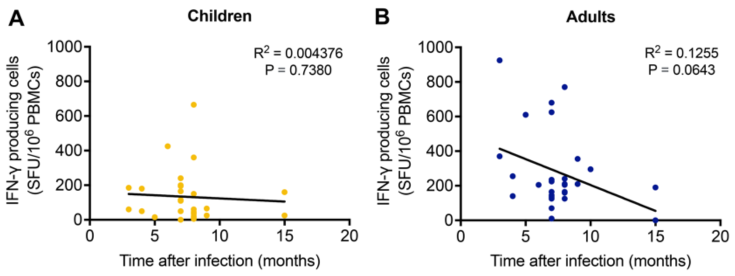

T-cell responses were assessed by ELISpot assay after peptide stimulation. Samples were collected from SARS-CoV-2 infected **(A)** children (N = 28, yellow) and **(B)** adults (N = 28, blue). **(A-B)** PBMCs were stimulated with peptide pools from the ancestral SARS-CoV-2 spike. Correlations between number of IFN-γ secreting cells and months since infection are shown. Correlations were assessed by Pearson correlation coefficient. Coefficient R^2 and P-values are shown.

Figure S6. Adaptive immune responses to SARS-CoV-2 were comparable in males and females

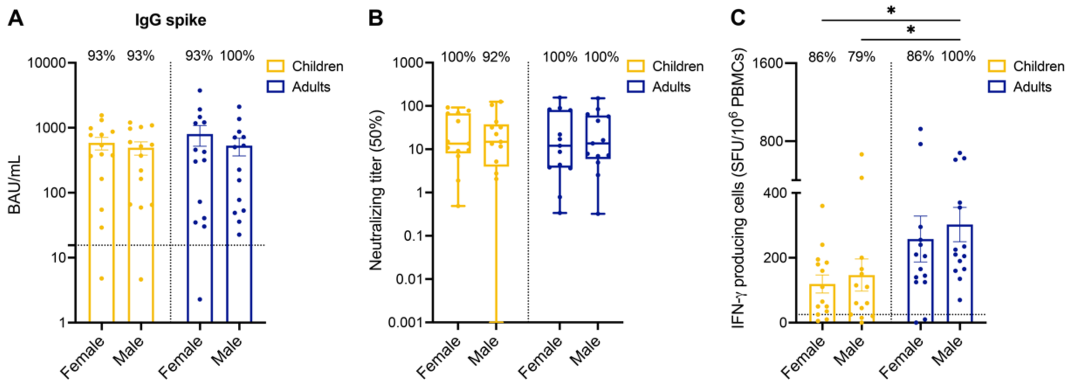

Samples were collected from equal numbers of children (N = 28) and adults (N = 28) from each sex (N = 14). **(A)** ELISA were conducted to measure the anti-spike IgG in the serum. The dotted line indicates the positive threshold value of 15.53 BAU/mL. The percentage of participants with IgG responses above the cut-off are shown for each group. **(B)** Surrogate neutralization ELISA were conducted to establish the serum dilution level (ID50) required to inhibit 50% of the binding between the trimeric spike protein and the ACE2 receptor, thus neutralizing the virus attachment capability to cells. The percentage of participants with detectable neutralizing antibodies against the ancestral strain are indicated for each group. **(C)** T-cell responses were assessed by ELISpot assay after stimulation with peptide pools from the ancestral SARS-CoV-2 spike. The dotted line indicates the positive threshold value of 25 IFN-γ secreting cells. The percentage of participants with values above this threshold are shown for each group. For each of the three assays performed, no sex-based difference were noted. Error bars indicate mean ± SEM. Statistical significance was established as not significant (ns), not shown on graphs P >.05, *P <.05.

Table S1. Statistical analyses for sex-based comparisons of SARS-CoV-2-specific adaptive immune responses

Antigen or Viral Strain		P-values between female and male in children	P-values between female and male in adults
**ELISA on serum**			
IgG	Spike	0.8036	0.9459
	RBD	0.9100	0.8388
	Nucleocapsid	0.7345	0.5407
IgA			
	Spike	0.1488	0.7584
	RBD	0.1151	0.5395
	Nucleocapsid	0.7636	0.8383
**Surrogate neutralisation ELISA on serum**			
Ancestral (Wuhan-like)		0.8743	0.9598
Omicron BA.1		0.5409	0.5446
Omicron BA.4/BA.5		0.9100	0.9506
**ELISpot on PBMCs**			
Spike - Ancestral		0.9010	0.3341
Nucleocapsid - Ancestral		0.6571	0.3950
Membrane - Ancestral		0.9517	0.5263
Spike - Alpha		0.5416	0.3403
Spike - Beta		0.7648	0.1782
Spike - Gamma		0.6221	0.3212
Spike - Delta		0.9904	0.0591
Spike - Omicron		0.9008	0.1245
Spike - HCoV-OC43		0.9795	0.0916
Spike - HCoV-HKU1		0.9463	0.8694
